# Neighborhood social environment and mental health of older adults in China: the mediating role of subjective well-being and the moderating role of green space

**DOI:** 10.3389/fpubh.2024.1502020

**Published:** 2024-12-06

**Authors:** Taizhi Lin, Qianhui Wang, Zixuan Tan, Wen Zuo, Rong Wu

**Affiliations:** ^1^Guangzhou Urban Planning and Design Company Limited, Guangzhou, China; ^2^School of Architecture and Urban Planning, Guangdong University of Technology, Guangzhou, China

**Keywords:** mental health, social environment, subjective well-being, green space, older adults

## Abstract

**Introduction:**

With the continuous development of the global aging trend, the mental health of older adults has been a concern by the world. The living space of older adults is limited due to the decline of their activity function. Neighborhood environment, especially the neighborhood social environment, has become an important factor affecting the mental health of older adults. Therefore, this study explores the mechanism that influences the social environment of the neighborhood and the mental health of older adults, the mediating effect of subjective well-being (SWB), and the moderating effect of green space.

**Methods:**

Based on the 2018 China Labor Dynamics Survey, this study used the structural equation model to explore the mediating effect of neighborhood social environment (neighborhood ties, social trust, community security) on the mental health of older adults through SWB and the moderating effect of green space.

**Results:**

Social trust and community security are both directly and positively associated with older adults’ mental health. At the same time, neighborhood ties, social trust, and community security can promote the mental health of older adults by positively affecting SWB, while green space has an enhanced moderating effect between neighborhood ties and mental health.

**Discussion:**

This study enriches the empirical research on neighborhood social environment and mental health. First of all, older adults living in communities with good safety conditions and high social trust are less affected by negative emotions and tend to have good mental health. Second, deeper neighborhood ties, higher social trust, and safer community environments help older adults to be less disturbed by negative situations, have a positive effect on their SWB, and indirectly promote mental health. At the same time, green space can provide a place for older adults to socialize, enhance the positive impact of neighborhood ties on SWB, and further promote the mental health of older adults. Finally, this study suggests that the government and community managers pay attention to the construction of neighborhood social environment and green space, and provide support for “healthy community” and “healthy aging” planning.

## 1 Introduction

The aging population presents a global challenge to social and economic growth. Compared to younger adults, emotional expression and communication channels constitute the primary spiritual needs of older adults owing to functional decline and physical disease; therefore, mental health problems caused by aging have gradually attracted increasing attention (1, 2). The World Health Organization (WHO) has pointed out that the mental health issues of older adults increase as their mental health declines over time. Suicide rates are higher among older adults than among teenagers and middle-aged persons; the rate of suicide in this population reached 27.2% in 2019 (3). In addition, spaces for daily activities and social interaction for older adults shrink as they age (4). This also stems from older adults’ lack of knowledge about potential mental health risks. Emerging economies may face more complicated risks of population aging owing to the imbalance between population growth and economic development. China is predicted to have a deeply aging population by 2036–2050. Moreover, the neighborhood environment, as the main location for activities and a source of social interaction, can influence the health of older adults (4–6). In this context, we must consider the importance of neighborhood social environment, including neighborhood ties (7, 8), social trust (9, 10), community security (11, 12), neighborhood greenness (13, 14), and other factors. Neighborhoods with high population density and land diversity enable residents to have more routes or destination choices for leisurely activities and build close community ties; this encourages people to participate in group exercises, which can help reduce the risk of depression (15, 16). People who enjoy communicating with family and neighbors and who actively participate in community group activities and social interactions tend to have a positive mental state (17). Wang et al. hold that with improvements in community security, residents are more likely to trust their neighbors and communicate with them frequently (18).

Previous studies have focused on how the neighborhood environment in general affects the mental health of older adults. However, there are few studies on the potential mechanism of neighborhood environment affecting the mental health of older adults, especially the mediating effect of neighborhood social environment on the mental health of older adults and the moderating effect of various factors (19, 20). Thus, this study aimed to systematically examine the relationship between the neighborhood social environment and the mental health of older adults and further explored the important moderating and mediating pathways between them. Our conclusions aim to optimize urban construction and implement construction strategies for a healthy environment to create healthy aging communities and age-friendly cities.

## 2 Literature review

### 2.1 Research on neighborhood environment and mental health of older adults

According to the WHO, mental health is generally defined as a state of well-being in which an individual realizes his/her abilities, copes with the normal stresses of life, works productively, and can contribute to his/her community (21). It encompasses emotional, psychological, and social well-being, influencing how individuals think, feel, and behave across different stages of life (22). In older adults, mental health is particularly important, as it significantly affects quality of life, functional independence, and overall well-being. This highlights the need for targeted interventions that promote positive mental health and prevent related issues in this age group (23). As an abstract concept, mental health is often assessed using multiple indicators, which are identified through both subjective and objective approaches. Internationally recognized scales that are frequently used include the Symptom Checklist-90 (SCL-90), the Positive and Negative Affect Schedule (PANAS), the Center for Epidemiological Studies Depression (CES-D) Scale, and the Self-Rating Depression Scale (SDS). At the same time, advances in technology have enabled digital health tools, including smartphone applications and wearable devices, to play an increasing role in real-time mental health monitoring (24).Among them, the CES-D-20 is a reliable, valid, easy-to-administer tool that is sensitive to changes in depressive symptoms, making it ideal for both large-scale studies and clinical settings (25–28). Thus, we derived our information from questionnaire data of the 2018 China Labor Dynamics Survey (CLDS), in which the assessment of mental health was based on the CES-D Scale as a reference. Currently, research on the mental health of older adults revolves around four main pathways: individual factors (e.g., medical history, age, gender, habits) (29, 30), family factors (31, 32), community factors (33), and aging care services (34, 35). Different family structures have varying effects on the mental health of older adults. Important social relationships with family can have both positive and negative health effects, depending on the quality, frequency, and strength of that connection (36). Moreover, residents’ subjective perceptions of the neighborhood environment have a strong impact on their well-being and health status. People who live in a cohesive community can obtain information and support from their neighbors, thereby benefiting their mental health (37, 38). In addition, with the decline in physical function and mobility as well as the shrinking of social networks, older adults’ dependence on the community increases with age. Hence, neighborhood ties in the community help to alleviate loneliness and anxiety in older adults and provide emotional value through neighborhood interactions (39). Further, with the community being the basic unit of China’s current social governance, the older adult care model is rooted in home-based care, supported by community-based care, and supplemented by institutional care. Community-based care for older adults is more effective at mitigating physical and mental health problems caused by aging (40, 41).

To date, numerous studies have examined the effects of neighborhood environments on mental health. From the standpoint of person-environment fit theory, the neighborhood environment mostly affects older adults’ mental health through interactions involving functional ability (42, 43). Some studies suggest that although the functioning of older adults may have declined, the neighborhood environment meets their social needs and enhances their sense of belonging. This helps reduce loneliness and social anxiety in older adults, which positively affects their mental health (44). Neighborhood environments primarily include social and built environments. The social environment reflects interactions between neighbors, which encourages older adults to participate in social interactions and disseminate and share information. Neighbors with abundant facilities offer them the opportunity to communicate, which improves active social interaction and formulates excellent neighborhood ties (45, 46). Liu et al. emphasized the important role of the social environment in neighborhood attachment and alleviating feelings of exclusion and isolation (40); they asserted that a cohesive, supportive community provides neighborhood support to residents and reduces stress in their lives. Stressors inside socioeconomically deprived neighborhoods (such as anti-social behavior or environmental disorders) stimulate negative emotions, thus influencing mental health. Baranyi et al. proposed that living in a high-crime neighborhood may directly or indirectly impact mental health (47). Social trust can promote engagement with social networks, thereby improving mental health (48). Moreover, individuals who interact with trustworthy neighbors or are willing to help their neighbors may develop a positive psychological state by acquiring a sense of security and acceptance within the community and by recognizing their self-worth (49, 50).

The built environment is defined as an objective material setting constructed by humans for living and production activities in cities (51). Thus far, several studies have been grounded in the “5Ds” theory, which holds that density (e.g., building density, population density, etc.), diversity (e.g., diverse leisurely activities), design (e.g., sidewalk coverage, street trees, average street widths, etc.), destination accessibility (e.g., points of interest, accessibility to the nearest parks and squares, etc.), and distance to transit (e.g., the distance between transit stops) are significantly associated with the mental health of older adults (52–54). Under the current development trend of urban spatial agglomeration, scholars are paying more attention to and finding evidence of a positive relationship between green space and mental health. Urban greenness provides a safe, attractive, and accessible walking place for surrounding residents, potentially alleviating depressive symptoms (55). Green spaces, such as parks and gardens, have been widely studied for their positive effects on mental health. Research suggests that exposure to green spaces can reduce stress, enhance mood, and improve overall mental well-being (56, 57). These spaces offer restorative environments, encouraging relaxation and physical activity, which are beneficial for both cognitive function and emotional regulation (58). In older populations, the mental health benefits of green spaces are particularly significant. Older adults often face increased risks of depression and cognitive decline due to reduced social interaction and physical activity. Green spaces can mitigate these risks by providing opportunities for social engagement, exercise, and sensory stimulation (59). Walking in parks or spending time in green areas has been linked to lower levels of anxiety and depression among older individuals, improving their overall quality of life (60). Moreover, studies have shown that accessibility and proximity to green spaces are crucial for older populations, as these factors determine the likelihood of frequent visits and engagement (61). Urban planning initiatives that increase access to green areas have the potential to promote healthier aging by supporting both mental and physical health in older adults. Additionally, green spaces contribute to a sense of community, helping alleviate feelings of isolation, which is a common challenge among older adults (62). Furthermore, satisfaction with neighborhood green space encourages people to use green areas more frequently, resulting in greater esthetics, pleasure, and relaxation (63). At the same time, some scholars believe that there are potential variables in the moderating and mediating roles between them.

### 2.2 SWB and mental health

The mental health of older adults has become a significant issue investigated in the fields of health psychology, genealogy, and other areas (64). In light of the recent strides in positive psychology, SWB has turned into one of its crucial contents (65). Studies have shown that is an important index to measure the mental health and life quality of older adults. Factors affecting SWB are subjectivity, stability, and wholeness (66). Research indicates that long-term physical health problems often lead to mental health problems like depression and anxiety, reducing SWB and quality of life under the conditions of China’s rapid aging (67). With regard to mental health, it not only prevents depression but also contributes to SWB. For instance, people with better mental health can have optimistic attitudes that enable them to cope effectively with life’s adversities and challenges, resulting in higher SWB. As for SWB, generally speaking, people with higher levels of SWB are found to lead a healthier life or live longer (68). The possibility is that positive SWB is a protective factor for health. Furthermore, prospective-epidemiological research suggests that positive life evaluations and hedonic states such as well-being predict lower future mortality and morbidity (69). In addition, SWB and mental health are closely linked to age while their relation is probably bidirectional which means SWB and mental health interrelate. Additionally, according to Baird’s point, the SWB follows a U-shaped trajectory, rising with age before declining, notably after 70 years (70).

**Figure 1:**
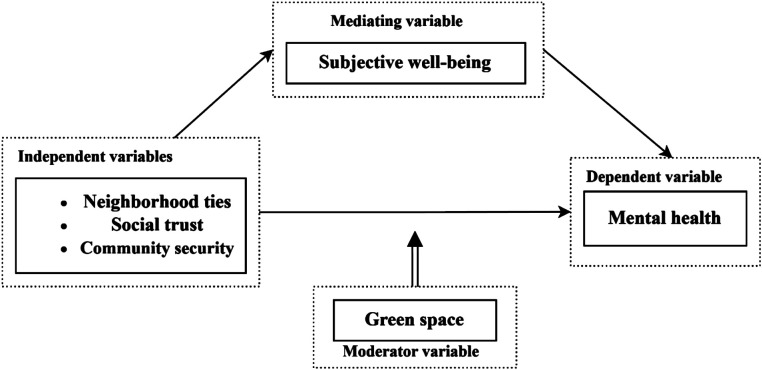
Theoretical framework for the SEM construction.

Neighborhood environments have been identified as being relevant to promoting human health and enhancing well-being (71). Studies have shown that SWB as a crucial indicator is used for evaluating residents’ well-being, and Huppert et al. considered happiness to measure the characteristics of residents and communities (72). On this basis, the built environment and the social environment are two domains of neighborhood context that are related to mental health. In terms of the built environment, green space is a vital factor, and there is growing evidence that green space is beneficial for mental health. Especially among vulnerable groups (e.g., older adults), green space in cities can be associated with improved overall well-being and self-perceived health status, suppressed morbidity and increased life expectancy, and increased satisfaction with life prospects, among other ways to promote healthy aging in older adults (73). Green space not only provides a place for social activities and physical activities for older adults, but also its rich natural landscape can reduce loneliness and improve emotional health, thus promoting SWB (74). As for the social environments, neighborhood social environment such as community security, social trust, and other factors have a great influence on people’s interaction in the community, which plays a vital role in mental health and well-being. According to Ballas and Tranmer, the neighborhood with a high-security situation can often improve the SWB of individuals (75). Meanwhile, neighborhood ties lead to the creation of a friendly neighborhood atmosphere with high levels of trust and reciprocity, protecting residents from pathological mental states such as depression and anxiety which contributes to SWB (76). Although no study has clearly demonstrated the mediating role of SWB, most research approves of the viewpoint that neighborhood factors are significantly related to daily life, mental health, and SWB (53, 77). SWB has the potential to be an intermediate variable among these variables (13). Based on this, this study aims to explore pathways linking neighborhood social environment (neighborhood ties, social trust, and community security) to older adults’ mental health in the Chinese context. In the meanwhile, it particularly investigates the extent to which SWB mediates the linkage between neighborhood social environment and older adults’ mental health (Figure 1). What is more, it further explores the moderating role of green space and puts forward the following hypotheses:

*Hypothesis* 1: Neighborhood ties, social trust, and community security directly affect the mental health of older adults.

*Hypothesis* 2: Neighborhood ties, social trust, and community security affect the mental health of older adults by mediating SWB.

*Hypothesis* 3: Green spaces moderate the association between neighborhood ties, social trust, community security, and the mental health of older adults.

*Hypothesis* 4: Green spaces moderate the mediation of neighborhood ties, social trust, and community security on the mental health of older adults through the mediator of SWB.

## 3 Research design

### 3.1 Study area and dataset

**Figure 2:**
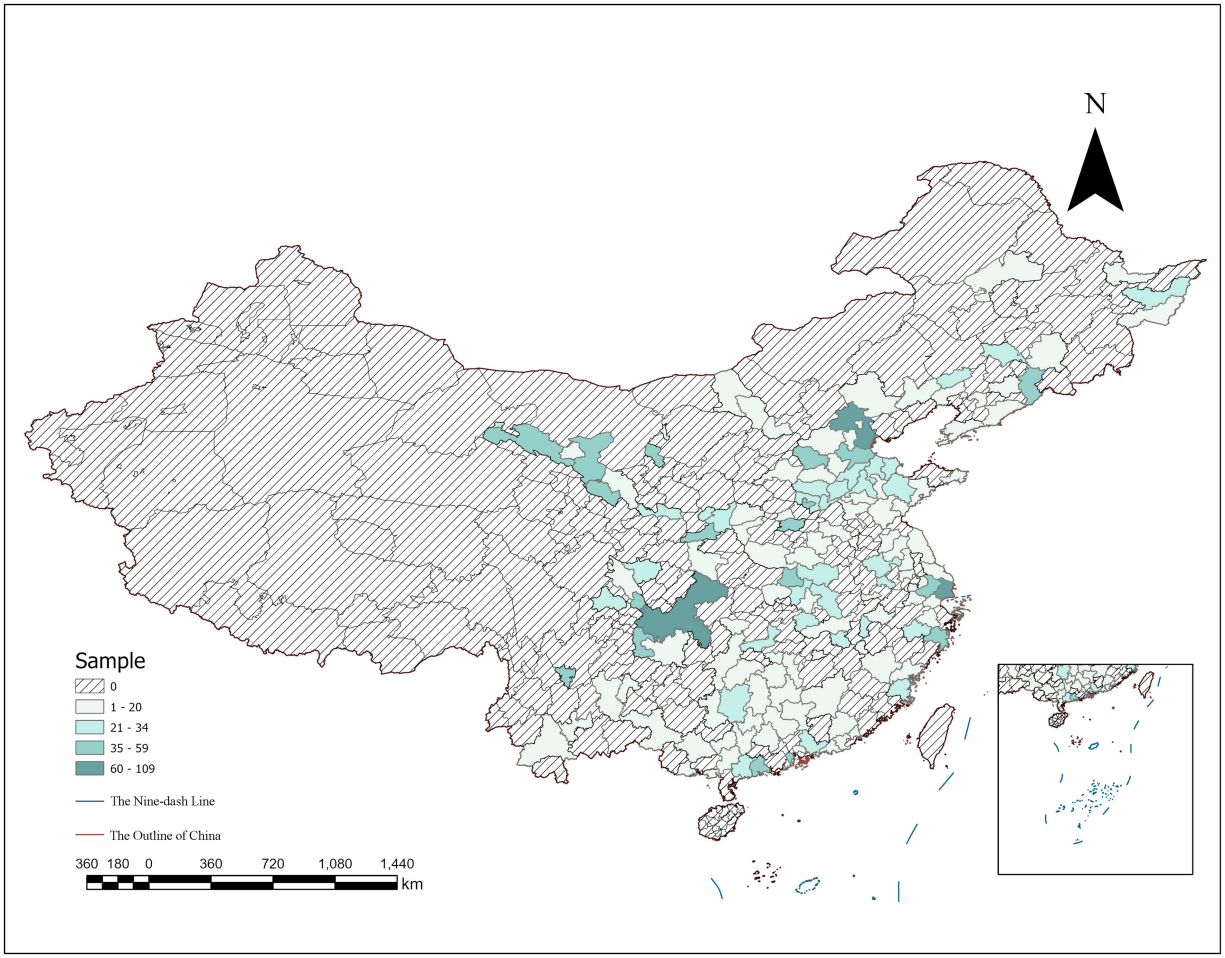
Distribution map of sample urban across the country. Based on the Department of Natural Resources Standard Map Service website GS(2019)18224. Standard maps are produced with no modifications to the base map boundaries, same as below.

Data for this study came from the 2018 CLDS, a large-scale, nationally representative tracking survey of labor force dynamics designed and implemented by the Center for Social Science Research at Sun Yat-sen University. The 2018 CLDS involved data gathered from 28 provinces in China, excluding Hong Kong, Macao, Taiwan, Tibet, Hainan, and Xinjiang. The database contains comprehensive data on 368 communities, 13,501 households, and 16,537 individuals in the labor force. The 2018 CLDS adopted multistage, multilevel probability sampling proportional to the size of the labor force, which minimizes sampling errors and ensures the randomness and scientific nature of sample selection. This study included men and women aged 60 and 55 years, respectively. The final sample comprised 3,315 individuals from 255 communities across 26 provinces (Figure 2).

Table 1 presents the descriptive statistics for all variables. The mean mental health score was 71.91 (SD ± 9.19), which is much higher than the cutoff (2/3 of the total score of 80), indicating that the participants had good mental health. The mean SWB score was 10.95 (SD ± 2.33), which is above the threshold, suggesting that the participants had a high level of SWB. The average levels of neighborhood ties, social trust, and community security were 7.66 (SD ± 1.58), 30.65 (SD ± 4.18), and 21.74 (SD ± 3.53), respectively. In terms of covariates, 59.4% of the respondents were female, 91.2% were not single, and 15.1% had suffered an illness or injury in the past 2 weeks. Their average age was 67.69 years old, and their average annual income in 2017 was 17,333.85 yuan.

**Table 1:** Statistics of variables.

Variables	Assignments	Total (*N* = 3,315)
Dependent variables		
Mental health [mean (SD)]	Continuous variables (20–80)	71.91(9.19)
Independent variable		
Neighborhood ties [mean (SD)]	Continuous variables (2–10)	7.66(1.58)
Social trust [mean (SD)]	Continuous variables (9–45)	30.65(4.18)
Community security [mean (SD)]	Continuous variables (5–25)	21.74 (3.53)
Mediators		
Subjective well-being [mean (SD)]	Continuous variables (3–15)	10.95 (2.33)
Moderator variable green space [mean (SD)]	Continuous variables	41.16 (3.66)
Covariates		
Annual personal income (mean)	Continuous variables	17,333.85
Marital status [N (%)]	1 = Non-single	3,023(91.2%)
	0 = Single	292(8.8%)
Age (mean)	Continuous variables	67.69
Gender [N (%)]	1 = Male	1,347(40.6%)
	0 = Female	1968(59.4%)
Sickness and injury status [N (%)]	1 = No sickness or injury within the last 2 weeks	2,816(84.9%)
	0 = Sickness and injury within	499(15.1%)

### 3.2 Variables and measurement

#### 3.2.1 Independent variables: neighborhood social environment

*Neighborhood ties*. Based on Hays et al., Liu et al., Dang et al., the measurement of neighborhood ties includes indicators such as neighborhood interactions, mutual assistance, neighborhood trust, neighborhood friendship, and community connections (78–80). This study considered older adults as the research object, focusing on their familiarity with the community and the frequency of mutual assistance. Neighborhood familiarity is measured via the question, “How familiar are you with your neighbors and other residents in your community?” A 5-point scale is used to provide an answer (1 = *very unfamiliar*, 2 = *not very familiar*, 3 = *generally familiar*, 4 = *relatively familiar*, and 5 = *very familiar*). Neighborhood mutual assistance is measured via the question, “Do you help your neighbors and other residents in your community (village)?” A 5-point scale is used to provide an answer (1 = *very little*, 2 = *relatively little*, 3) *in general*, (4) *relatively more*, and (5) *a lot.* The above items were summed to obtain the total score to generate the indicator of neighborhood ties. The overall scale score ranges from 2 to 10, and the higher the scale score, the better the neighborhood ties experienced by the respondent.

*Social trust*. This study assessed social trust via the question, “How much do you trust people in the following nine categories?” The categories include family members, relatives, friends, neighbors, classmates, strangers, people who work or do things together, businessmen who buy things, and people with religious beliefs. This study determined social trust scores using a 5-point Likert scale (1 = *not trustworthy* at all; 5 = *totally trustworthy*). The total score ranged from 9 to 45, with higher scores suggesting deeper levels of social trust. The Cronbach’s *α* of the social trust subscale was 0.74, implying that the questionnaire had reliability.

*Community security*. This study evaluated community security using the following question: “Within this community, do you have any of the following concerns?” The categories include the safety risk of hanging out, the safety risk of going out alone at night, the risk of burglary if one does not lock one’s doors and windows, the risk of being targeted after exposing one’s money, and the risk of one’s children being trafficked when they are out alone. This question is answered using a 5-point reverse scale (1 = *never*; 5 = *often*). The total score ranges from 5 to 25, with higher scores implying a greater sense of community security. The Cronbach’s *α* of the community security subscale was 0.816, indicating that the questionnaire had reliability.

#### 3.2.2 Dependent variable: mental health

This study employed the CES-D Scale to assess mental health; it contains 20 items used to evaluate depressive symptoms (81) and is scored on a 4-point reverse scale (1 = *almost always or 5–7 days per week*; 2 = *often or 3–4 days per week*; 3 = *rarely or 1–2 days per week*; and 4 = *never or < 1 day per week*). The total score ranges from 20 to 80, with higher scores suggesting improvement in the mental health of older adults compared to the previous week. The Cronbach’s *α* for the mental health subscale was 0.946, meaning the questionnaire was reliable.

#### 3.2.3 Mediator: SWB

At present, one of the common ways to measure subjective well-being (SWB) is the life satisfaction orientation, which includes both general and specific assessments of happiness and life satisfaction (82). Life Happiness, Life Satisfaction are recognized as key indicators for measuring SWB (83). Life Happiness has demonstrated significant advantages in assessing emotional experiences, capturing short-term feelings of joy or contentment (84). Life Satisfaction is commonly used for the subjective evaluation of overall life quality and long-term well-being (85). Furthermore, Economic Satisfaction has proven to be a significant predictor of SWB, effectively capturing the impact of economic circumstances on individual well-being (86). Ouyang et al. also used similar indicators of life happiness, life satisfaction and economic satisfaction to measure SWB (87). Therefore, this study uses the “Happiness Scale” in the questionnaire of CLDS database to measure SWB of individuals, which mainly includes three measurement items: “life happiness, life satisfaction and economic satisfaction.” This study measured happiness in life via the question, “Do you think you are living a happy life?” This study measured life satisfaction using the question, “Are you satisfied with your life?” based on the Measurement of Life Satisfaction Scale. This study measured financial satisfaction via the question, “Are you satisfied with your family’s financial situation?” The answers are rated on a 5-point Likert scale. This study summed up the scores of the three items to form an SWB index. The total score for the three items ranges from 3 to 15 points. The higher the score, the greater the SWB of older adults. The Cronbach’s *α* of the SWB scale was 0.824, suggesting that the questionnaire had reliability.

#### 3.2.4 Moderator: green space

Referring to existing studies that use the proportion of green space to measure green space (88), this study employed the coverage rate of green space in built-up regions as an index to quantify UGS and study the moderating effect. The greenspace coverage rate of built-up regions refers to the share of urban built-up areas covered by greenery to the total built-up area obtained from the 2018 China Urban Statistical Yearbook (89). Owing to the random treatment of communities in the questionnaire, this study could not locate each respondent’s community but rather only the city information related to each respondent. As such, this study assigned values according to the city information related to each respondent and each city’s greenspace coverage rate.

#### 3.2.5 Covariates

This study adjusted the study for covariates of older adults’ sociodemographic and individual health characteristics (90). For individual-level covariates, this study included gender (binary variable: male vs. female), marital status (binary variable: not single vs. single), and annual individual income (continuous variable). For covariates of individual health characteristics, this study used disease and injury status indicators (binary variables: no disease or injury in the past 2 weeks vs. illness and injury within the past 2 weeks). As the mental health of older adults may be affected by chronic disease, as there were no chronic disease-related problems in the CLDS in 2018, the illness and injury conditions of older adults could replace this problem to a certain extent (91).

### 3.3 Methods

This study used SEM to examine the mediating and moderating effects of neighborhood ties, social trust, community security, and the mental health of older adults in a neighborhood social environment. Notably, SEM can measure the total, direct, and indirect effects of one variable (such as neighborhood ties) on another (such as mental health) to explore the mechanisms underlying the ties between the neighborhood social environment and mental health in the community (92). This study tested the chain-mediation model using SEM. In the baseline model, mental health, SWB, neighborhood ties, social trust, and community security are continuous variables. In this study, green space, socio-demographic characteristics, and personal health characteristics were taken as exogenous variables, while neighborhood ties, social trust, community security, SWB, and mental health were taken as endogenous variables. At the same time, considering the possible collinearity between mental health and SWB variables, we used SPSS to conduct a collinearity test. The test results show that the VIF value between variables is less than 2 and the tolerance greater than 0.1, which alleviates the collinearity problem between variables. Additionally, we considered existing research to determine the fit parameters for SEM (93), which allowed us to test the proposed models. We used the following parameter criteria for model fit: the root mean square error of approximation (RMSEA) ≤ 0.05; the goodness-of-fit index (GFI) ≥ 0.90; the normed fit index (NFI) ≥ 0.80; the incremental fit index (IFI) ≥ 0.80; the Tucker-Lewis index (TLI) ≥0.80; and the comparative fit index (CFI) ≥ 0.80. We employed SPSS Amos 26 for SEM and STATA version 13.1 for basic pre-analysis data cleaning.

## 4 Results

### 4.1 Relation between neighborhood social environment and mental health of older adults

This study uses SEM models to investigate the association between neighborhood ties, social trust, community security, and mental health of older adults in the neighborhood social environment, exploring the mediating role played by SWB in this association, while adjusting for control variables at the sociodemographic and personal health levels of older adults. The fitting parameter criteria for the mediation effect model with moderation are shown in Section 3.3. The model path coefficients are shown in Table 2. Regarding sociodemographic characteristics control variables, older adults with younger age, higher annual income, and male gender were more likely to report better mental health status. At the level of personal health, older adults who have not been injured in the past 2 weeks are more likely to have good mental health. The direct path coefficient from social trust to the mental health of older adults is significant and positively correlated (*β* = 0.155, *p* < 0.05), which indicates that the improvement of social trust of older adults is helpful in promoting their mental health level. At the same time, the sense of community security of older adults also has a positive and direct impact on their psychological health (*β* = 0.098, *p* < 0.01). Therefore, Hypothesis 1 is partially supported. Additionally, there is a positive correlation between neighborhood ties, social trust, community security, and SWB. Closer neighborhood ties contributed to the improvement of SWB in older adults (*β* = 0.141, *p* < 0.01). Moreover, the improvement of older adults ‘s social trust also helps to maintain better SWB (*β* = 0.272, *p* < 0.01). A good sense of community security had a positive effect on the SWB of older adults (*β* = 0.039, *p* < 0.01). Furthermore, Older adults with better SWB also have a direct and positive impact on their mental health status (*β* = 0.445, *p* < 0.01).

**Table 2:** Model path coefficient diagram.

Path	Estimate	S.E.	C.R.	*p*
Neighborhood ties → mental health	0.007	0.039	0.184	0.854
Social trust → mental health	0.155	0.061	2.541	**
Community security → mental health	0.098	0.017	5.778	***
Neighborhood ties → SWB	0.141	0.029	4.943	***
Social trust →SWB	0.272	0.047	5.781	***
Community security → SWB	0.039	0.012	3.171	***
Age → mental health	−0.008	0.003	−2.297	**
Marital status → mental health	0.059	0.057	1.024	0.306
Gender → mental health	0.162	0.036	4.505	***
Annual income → mental health	0.056	0.009	6.209	***
Injury status → mental health	0.411	0.045	9.065	***

SWB, subjective well-being; *All the coefficients are standardized regression coefficients. *p < 0.1, **p < 0.05, ***p < 0.01.

### 4.2 The mediating effect of SWB

**Figure 3:**
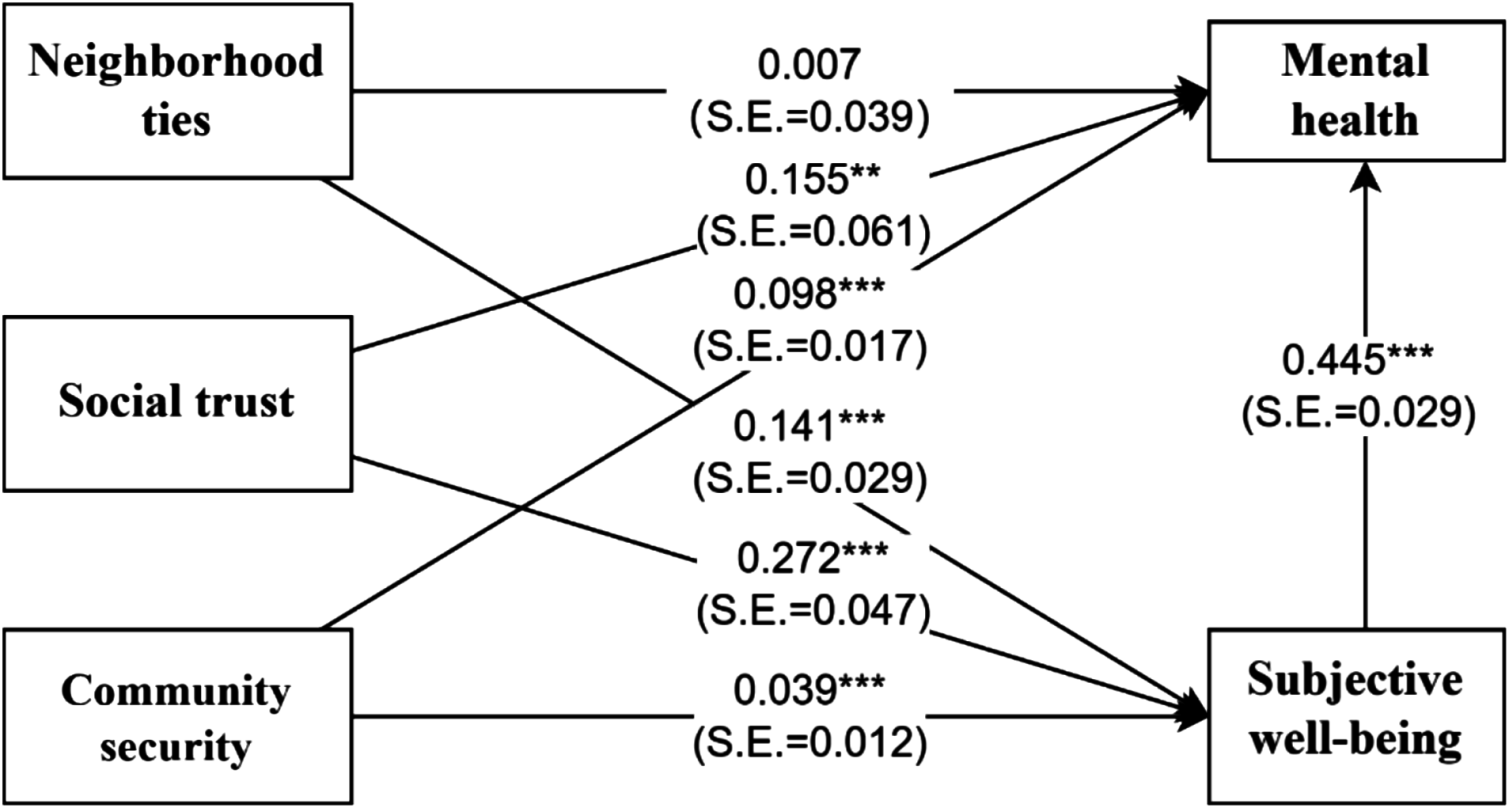
Structural equation modeling of the mediating role of subjective well-being among neighborhood relationships, social trust, community security, and mental health status. *p < 0.1, **p < 0.05, ***p < 0.01.

Table 3 and Figure 2 present the results of the mediating effects. Among the mediating effects of neighborhood social environment-related indicators on mental health, neighborhood ties, social trust, and community security were all significant and positively correlated with the mental health path coefficients of SWB through the mediating variables (neighborhood ties: *β* = 0.061, *p* < 0.01; social trust: *β* = 0.127, *p* < 0.01; community security: *β* = 0.017, *p* < 0.01). Thus, Hypothesis 2 is supported. The neighborhood ties index showed a complete mediating effect. The higher the neighborhood ties index, the more frequent the contact and interaction between the individual and a neighbor, and the stronger the social bond. Such a connection is conducive to improving the SWB of older adults and thus has a positive impact on their mental health. Moreover, social trust and community security had partially mediating effects. Improving social trust in older adults can contribute to good mental health by promoting SWB. A high level of community security indicates that older adults are in a relatively safe community and are not troubled by the presence of community security, which also helps to improve their SWB and mental health (Figure 3).

**Table 3:** Mediation effect test results.

Path		Estimate	Bootstrapping				S.E.	*p*
			Bias-corrected		Percentile			
			Lower	Upper	Lower	Upper		
Direct effect	Neighborhood ties → mental health	0.007	−0.068	0.087	−0.070	0.085	0.040	0.813
	Social trust → mental health	0.155	0.030	0. 295	0.029	0.293	0.066	**
	Community security → mental health	0.098	0.060	0. 133	0.061	0.133	0.018	***
Indirect effect	Neighborhood ties → SWB → Mental health	0.063	0.035	0.096	0.034	0.094	0.015	***
	Social trust → SWB → Mental health	0.121	0.075	0.179	0.073	0.176	0.027	***
	Community security → SWB → Mental health	0.017	0.006	0.030	0.006	0.030	0.006	***
Total effect	Neighborhood ties → mental health	0.070	−0.014	0.156	−0.014	0.156	0.043	0.107
	Social trust → mental health	0.276	0.150	0.428	0.149	0.427	0.071	***
	Community security → mental health	0.115	0.078	0.151	0.078	0.152	0.019	***

SWB, subjective well-being; *All the coefficients are standardized regression coefficients. *p < 0.1, **p < 0.05, ***p < 0.01.

### 4.3 The moderating effect of green space

We examined the moderating effect of green space on the mediating effect of the neighborhood social environment and the mental health of older adults. Table 4 shows the results of this model, which indicate that green space had no moderating effect on the direct relationship between neighborhood ties, social trust, community security, and the mental health of older adults. As for the mediating effect, we found that green space had a moderating effect on the mediating effect of neighborhood ties and mental health (*β* = 0.012, *p* < 0.01). Hence, Hypothesis 4 is partially supported. The improvement in the greenspace index helped enhance the positive, indirect correlation between neighborhood ties and mental health through SWB. Green Spaces not only play a role as places where ties are initially established but also where they are re-established. As leisure spaces, green spaces provide opportunities for social interaction, thereby contributing to developing new social connections and strengthening existing ones, thereby promoting neighborhood ties (94). Favorable and intimate neighborhood ties can enhance SWB. The improvement of green space coverage may provide a more possible social interaction space for the close neighborhood ties of older adults, enhance their SWB, and further promote the improvement of the mental health level of older adults. However, this study did not find that green spaces have a moderating effect on the indirect relationships between social trust, community security, and mental health. A possible explanation is that trust is promoted by the social function of green space (95). The measure of green space used in this study is green space coverage, which is insufficient in evaluating the social function of green space. Relevant studies also prove that the relationship between green space and trust is more likely to be related to the weekly use frequency and single-use duration (96). Secondly, the moderating effect of green space on community security and the mental health of older adults is not significant, which may be related to the complex relationship between green space and community security. To date, the evidence on the relationship between green Spaces and feelings of safety and crime is mixed. Some studies show that green space vegetation may provide convenience for crime (97), but other studies believe that green space vegetation can help reduce residents’ fear of crime and enhance their sense of security (98). In a more detailed study of the relationship between green space vegetation types and crime, a higher proportion of grassland was associated with a lower rate of crime only for areas with relatively low crime rates, while a higher proportion of woodland was associated with a lower rate of crime only for areas with relatively high crime rates (99). Therefore, many other factors may influence the relationship between green space and security, which could explain why the moderating effect of green space was not significant.

**Table 4:** Moderation effect test results.

Path	Estimate	Bootstrapping				S.E.	*p*
		Bias-corrected		Percentile			
		Lower	Upper	Lower	Upper		
GNT → mental health	0.000	−0.038	0.038	−0.037	0.040	0.020	0.151
GST → mental health	−0.012	−0.054	0.032	−0.058	0.028	0.022	0.632
GCS → mental health	0.000	−0.058	0.009	−0.058	0.009	0.017	0.978
GNT → SWB → Mental health	0.011	0.000	0.023	0.000	0.024	0.006	*
GST → SWB → Mental health	−0.005	−0.017	0.008	−0.017	0.007	0.006	0.474
GCS → SWB → Mental health	0.000	−0.010	0.011	−0.010	0.011	0.005	0.964

GNT, Interaction Terms of Green Space and Neighborhood ties; GST, Interaction Terms of Green Space and Social Trust; GCS, Interaction Terms between Green Space and Community Security; SWB, subjective well-being; *p < 0.1, **p < 0.05, ***p < 0.01.

## 5 Discussion

In the past 30 years, China’s rapid migration and urbanization processes have led to significant changes in the composition and characteristics of urban communities. Various empirical studies conducted in Western countries have reported significant impacts of neighborhood social environmental characteristics such as green space, neighborhood social trust, and neighborhood ties on residents’ mental health (100, 101). By facilitating neighborhood ties, neighborhood green space can contribute to the development of neighborhood social trust, which has proven to benefit people’s health (102). Nevertheless, the moderating role of the built environment (e.g., green space) in the influence of the social environment (e.g., neighborhood ties, social trust, and community security) on mental health has not been sufficiently considered. Besides, numerous scholars have conducted research on the relationship between SWB and mental health and concluded that the close connection between health and SWB grows with age (103). Whereas, considering SWB as the mediator in the relationship between neighborhood social environment and mental health has not been adequately explored in existing research. For the purpose of enhancing our understanding of the bond between them, we identify SWB as the mediator through which neighborhood social environment may affect older adults’ mental health. Additionally, we examined whether green space served as a moderator in the nexus between neighborhood social environment and mental health on the basis of using the SEM. Our results can serve as a valuable resource for urban planners and decision-makers, helping to improve the mental health of older adults and promoting healthy aging.

Previous studies conducted in Western countries that have explored the neighborhood environment and mental health indicate that the neighborhood environment primarily affects mental health by influencing the frequency of fitness activities and social interactions, while the neighborhood social environment affects the health-related behaviors of others (104). For instance, people who live in neighborhoods with less community security are prone to stress, and social chaos and unsafe neighborhoods affect their mental health (105). Neighborhood social attributes—including interaction, trust, civic engagement, and perceptions of community security—are determinants of residents’ mental health (106). A stable neighborhood enhances face-to-face interactions, strengthens participation among residents, provides a sense of consistency and belonging, and promotes emotional support and access to material resources when individuals are exposed to stressors (107). Our findings support these views, social trust, and community security were positively associated with mental health, whereas neighborhood ties did not directly affect the mental health of older adults. Evidence suggests that older adults are more likely to be confined to residential areas because of retirement and mobility problems. Attractive and safe neighborhoods diminish mental problems and negative emotions, evoke positive emotions, and lead to better conscious evaluation of life circumstances; conversely, people’s mental health is negatively impacted when they are exposed to environmental stressors, which lead to psychological stress, mental problems, and a greater likelihood of depression (106). As for social trust, Wang et al. deduced that social trust may benefit the mental health of older adults by providing a source of mutual connection and respect and by improving older adults’ sense of purpose in life (108). When it comes to neighborhood ties, our findings extend previous research by suggesting that it has no direct impact on the mental health of older adults. According to a study of different life stages, the increase of social ties can help relieve depression and anxiety, and the strongest influence of adolescents and adults, but without statistical significance for older adults. This may be because adolescents and adults are more inclined to develop their social ties, but older adults are more likely to prune their social networks based on their emotional experiences (109). Thus, the findings of this study may stem from their mutually conflicting features and negative effects on health. This finding concurs with the claim that the link between neighborhood social ties and mental health is highly variable and complex (37, 110).

Regarding SWB, previous studies suggest that the strong association between health and SWB increases with age in both developed and developing countries, while older adults with higher SWB live longer and healthier than those with lower SWB (103). This study further verified the role of SWB as the mediator in the relationship between the neighborhood social environment and mental health. The perceived neighborhood environment cannot only promote healthy physical activity and reduce the risk of chronic disease (such as obesity and cardiovascular disease) but also provide psychological recovery and spiritual release for older adults and improve their happiness, thus promoting their mental health (111). Our research puts forwards conclusion that in line with previous works, we provided unequivocal evidence of an association between social trust, community security, neighborhood ties and mental health among SWB. Firstly, the finding of our study is the indirect effect of social trust on the mental health of older adults through the mediation of SWB, which aligns with the results of an earlier research which demonstrated that older adults with higher SWB have a strong ability to cope with mental health risks and lessen the impact of social interactions on their mental health (112). In addition, the results of the mediation analysis showed that SWB plays a mediating role between community security and the mental health of older adults. Our results confirmed the insights of Cramm and Nieboer, who have understood that community-dwelling older people who perceive their neighborhood as very safe benefit more from solidarity among neighbors, resulting in higher SWB (39). Moreover, concerning neighborhood ties, current studies assume that ties with older people of the same age (which make it easier to obtain social support from others) induce faster recovery from fatigue and discomfort, which can indirectly affect SWB and further improve mental health (113). As expected, neighborhood ties affected the mental health of older adults through SWB. Social interactions weaken social anxiety and loneliness in older adults, leading to more positive emotions in this demographic, thereby benefiting their mental health.

Furthermore, the result of this study found that green space played a moderating role in the mediating effect of SWB on the neighborhood social environment and mental health. As part of the neighborhood environment, green spaces are closely tied to the daily lives of older adults and provide numerous social and ecological services, thus representing an important part of an age-friendly environment (114). Green spaces have been associated with promoting human health and enhancing well-being (115). For instance, watching plants for 5 min improved psychological relaxation in older adults; participating in gardening activities related to green spaces can also increase psychological relaxation in older adults (116). Our empirical results provide further insight into the remarkable role of green space in that it played a moderating role in the relationship between neighborhood ties and mental health, whereas social trust and community security did not achieve the desired outcomes. The improvement of green space promotes the positive influence of neighborhood ties on SWB, and then improves the mental health state of older adults. Initially, green space provides spaces for people to experience nature and encourages older adults to enter such areas for social and physical activity (117). Green spaces offer opportunities for neighborhood interactions among older adults, thereby increasing the possibility of communication within the community. Moreover, older adults who regularly socialize have higher SWB and better mental health (118). Subsequently, based on the Biophilia Hypothesis, people’s psychological health is associated with their relationship to nature (119). Owing to the scarcity of urban green space resources and the decline of older adults’ individual functions, it may be difficult for older adults living in urban areas to access green space. In contrast to older adults, the younger with stronger physiological functions and weaker demand for surrounding green space prefer a longer distance for physical exercise (120). Therefore, this may make access to natural green spaces more valuable and important for older adults. Contact with nature promotes the prosperity of private personal lives and public social lives. Those who are highly nature-connected may derive a sense of meaningful presence from their closeness with nature, which may promote well-being (121). Increased contact with nature through green spaces in older adults has a positive impact on emotions (122), contributing to more meaningful neighborhood connections and prosperous social lives, thus deepening neighborhood ties has a positive indirect impact on mental health by promoting SWB. Additionally, the Stress Recovery Theory suggests that natural environments, as restorative environments, can provide residents with opportunities to appreciate natural landscapes, thereby enhancing their ability to cope with stress and promoting individuals to recover faster from stress (123). Frequent interaction with neighbors has a positive effect on SWB (124). Having positive, non-difficult relationships helps reduce stress and promote well-being (125). Green spaces may provide an environment for individuals to relieve stress and socialize, which encourages individuals to have a more relaxed attitude toward neighborhood interactions and promotes an improvement in overall well-being. Simultaneously, green spaces may act as stress-relief amplifiers, enhancing the benefits of neighborhood relationships to improve the SWB of older adults better, thereby achieving higher levels of mental health. Regarding the role of green space in the relationship between neighborhood ties and mental health, our conclusion is consistent with most studies. In addition, green space contributes to an area’s livability, particularly in deprived urban neighborhoods; green space is viewed as “safe, secure, attractive, socially cohesive and inclusive, and environmentally sustainable” (126, 127). This is inconsistent with our findings, which may be because our data for measuring the green space index is the green space coverage rate of built-up areas, which makes the research findings different.

Our results are of great significance for promoting the construction of healthy and livable cities in China and the successful aging of older adults. First, the government, community organizations, and housing managers should pay more attention to the social and neighborhood connections of older adults, recognize their diverse social needs, and create more opportunities for them to connect with other residents in the community, such as by providing more conducive places for older adults to socialize and holding more abundant neighborhood activities to encourage interactions. Second, as important natural and social spaces in the community, the government and community organizations should pay attention to the construction of green spaces. This study recommends that relevant departments regularly maintain and improve green spaces and promote their positive factors to benefit older adults. In addition, regarding the direct and indirect beneficial effects of social trust on the mental health of older adults, this study suggests that the government and community organizations build a harmonious, friendly community environment, organize community activities and lectures, and deepen the understanding of older adults’ social context to enhance their social trust and promote the improvement of their mental health. Finally, regarding the importance of community security, the government should issue scientific and accurate community management regulations. Community management institutions should strictly implement community management and improve community security; these institutions regularly listen to community residents to receive their feedback on community services and safety measures according to their needs. Adjustments are later made to create a safe community environment.

This study has some limitations. To begin with, the data selected in this study is cross-sectional, and there may be missing variables or unobservable differences between individuals in the statistical collection of cross-sectional data. Moreover, owing to data limitations, this study did not consider other attributes of neighborhood green space (e.g., quality, usage frequency, duration, visibility, accessibility), which may have influenced the mental health of older adults. Furthermore, relying solely on the coverage rate of green space inadequately captures the accessibility and quality green spaces, which introduces bias into the findings. Future research should employ more precise indicators for a more accurate assessment of green space characteristics. Ultimately, although our research is based on older adults, this study did not consider the impact of different family structures and different age groups of older adults.

## 6 Conclusion

To sum up, this study used the SEM model and statistical data from the nationally representative 2018 CLDS database to study the relationship between neighborhood society, the built environment, and the mental health of older adults in the Chinese community. This study focused on exploring the indirect impact of neighborhood ties, social trust, and community security on the mental health of older adults through SWB while paying attention to the moderating effect of green space on the mental health of older adults. The results show that (1) in the neighborhood social environment, social trust and community security had a direct, positive impact on the mental health of older adults. (2) Neighborhood ties, social trust, and community security indirectly improved the mental health of older adults through their positive effects on SWB. (3) Green spaces reinforced the positive and indirect effects of neighborhood ties on the mental health of older adults through SWB. The results of this study further confirm the importance of neighborhood social and built environments for the mental health of older adults. As such, relevant government departments and community managers should pay attention to the living experiences of older adults in the community and promote the construction of aging-friendly communities to cope with the trend of population aging and promote the development of healthy aging.

## Data Availability

The original contributions presented in the study are included in the article/supplementary material, further inquiries can be directed to the corresponding author.
